# Supplementary material for: Effects of Incorporating Alkaline Hydrogen Peroxide Treated Sugarcane Fibre on The Physical Properties and Glycemic Potency of White Bread
Source: Foods. 2023 Mar 29;12(7):1460. doi: 10.3390/foods12071460 (PMC10094325; doi:10.3390/foods12071460)
Supplement: Supplementary file 1 [file foods-12-01460-s001.zip › foods-2239611-Supplementary.pdf]

Supplementary information:

Effects of Incorporating Alkaline Hydrogen Peroxide Treated Sugarcane Fibre on The Physical Properties and Glycemic Potency of White Bread

Authors:

Fatin Natasha Binte Abdul Halim [1], Afsaneh Taheri [1], Zawanah Abdol Rahim Yassin [1], Kai Feng Chia [1], Kelvin Kim Tha Goh [2], Suk Meng Goh [1] and Juan Du [1,*]

[1] Food, Chemical and Biotechnology Cluster, Singapore Institute of Technology, 10 Dover Drive, Singapore 138683, Singapore
[2] School of Food & Advanced Technology, Massey University, Private Bag 11222, Palmerston North 4410, New Zealand
* Correspondence: du.juan@singaporetech.edu.sg

Table S1. PMI for different methods used in different paper.

	Our method	Sangnark & Noomhorm [18]	Alencar et al. [57]
AHP treatment condition	12.6% sugarcane fibre, 0.5-5 h, washed twice.	1% sugarcane bagasse, 12 h	10% corn staver biomass, 1h
Process Mass Efficiency (PMI)	18.4	101 – 200	29.2 – 34.8

$$\text{Process Mass Intensity [58]} = \frac{\text{Total mass in process steps (kg)}}{\text{Mass of products (kg)}} \quad \text{Eq. (S1)}$$

Table S2. Parameters to determine particle size of samples.

Sample Identification	Sugarcane Fiber
Particle Type	Spherical
Material	Cellulose
Refractive Index	1.4683 [59]
Absorption Index	0.001
Dispersant	Water
Dispersant Refractive Index	1.33
Measurement Duration	Background: 10 s<br>Sample: 5 s
Sequence	5
Measurement Obscuration	5 – 15 %

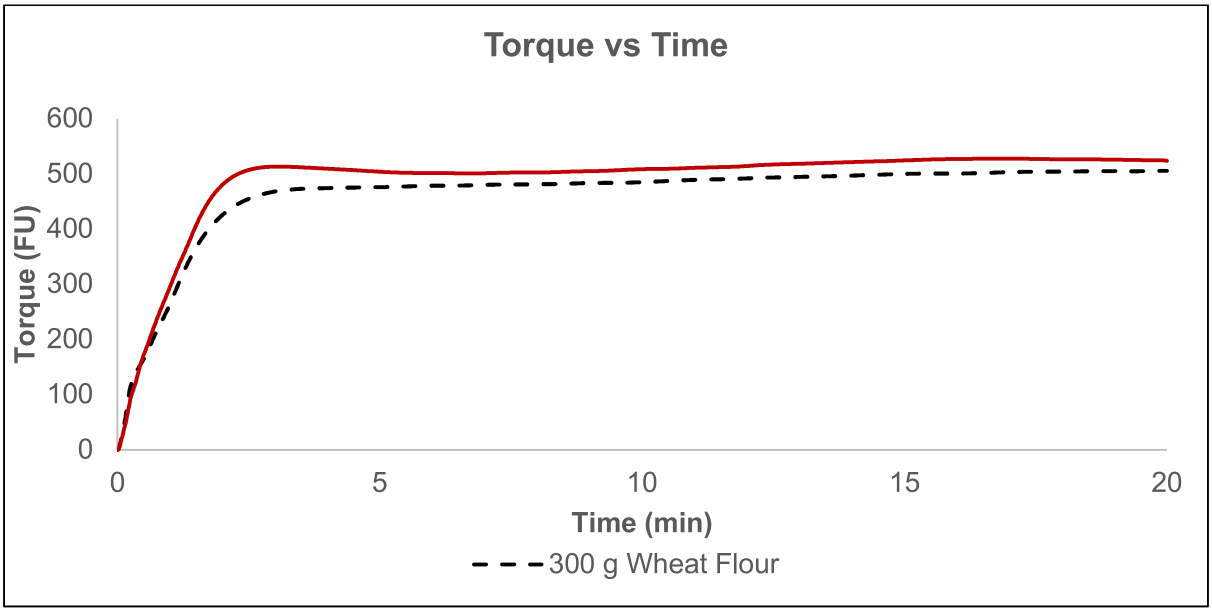

Fig S1. Farinograph analysis indicating the dough development of flour samples over a 20 min mixing regime.

Table S3. Dough extensibility test parameters on texture analyzer.

Parameters	Settings
Test Mode	Tension
Pre-Test Speed	2.00 mm/s
Test Speed	3.00 mm/s
Post-Test Speed	10.00 mm/s
Distance	90 mm
Trigger Force	5.0 g

Table S4. Double compression test parameters on texture analyzer.

Parameters	Settings
Test Mode	Strain – Double Compression Test
Pre-Test Speed	1.00 mm/s
Test Speed	2.00 mm/s
Post-Test Speed	2.00 mm/s
Initial Probe Height	40 mm
Trigger Force	5.0 g
Time Gap	5.0 s
Strain	40 %

Table S5. Total iAUC, relative glycemic potency (RGP), and rapidly digestible starch (RDS) values of bread samples.

Samples	Total iAUC (g.min)	RGP (g/100g bread)	RDS (%)
Reference	1431.4 ± 14.0^{a}	38.3 ± 0.3^{a}	43.6 ± 3.2^{a}
Untreated SCF	807.2 ± 35.4^{bc}	23.0 ± 0.9^{b}	28.3 ± 0.3^{bc}
0.5h AHP SCF	908.7 ± 25.4^{b}	22.4 ± 0.6^{bc}	30.3 ± 1.1^{b}
1h AHP SCF	753.6 ± 51.7^{c}	19.3 ± 0.6^{d}	24.3 ± 0.3^{c}
3h AHP SCF	704.0 ± 59.2^{c}	19.0 ± 1.0^{d}	23.4 ± 0.6^{c}
5h AHP SCF	723.1 ± 7.9^{c}	19.8 ± 0.1^{cd}	23.7 ± 0.6^{c}

References

57. Alencar, B. R. A., Reis, A. L. S., de Souza, R. D. F. R., Morais Jr, M. A., Menezes, R. S. C., & Dutra, E. D. (2017). Recycling the liquid fraction of alkaline hydrogen peroxide in the pretreatment of corn stover. Bioresource technology, 241, 928-935.
58. Monteith, E. R., Mampuys, P., Summerton, L., Clark, J. H., Maes, B. U., & McElroy, C.

R. (2020). Why we might be misusing process mass intensity (PMI) and a methodology to apply it effectively as a discovery level metric. *Green Chemistry*, *22*(1), 123-135. https://doi.org/10.1039/C9GC01537J.

59. Sultanova, N., Kasarova, S., & Nikolov, I. (2009). Dispersion properties of optical polymers. *Acta Physica Polonica A*, *116*(4), 585-587. http://dx.doi.org/10.12693/APhysPolA.116.585
